# Risk factor mining and prediction of urine protein progression in chronic kidney disease: a machine learning- based study

**DOI:** 10.1186/s12911-023-02269-2

**Published:** 2023-08-31

**Authors:** Yufei Lu, Yichun Ning, Yang Li, Bowen Zhu, Jian Zhang, Yan Yang, Weize Chen, Zhixin Yan, Annan Chen, Bo Shen, Yi Fang, Dong Wang, Nana Song, Xiaoqiang Ding

**Affiliations:** 1 Department of Nephrology, Zhongshan Hospital, Fudan University, Shanghai Clinical Research Center for Kidney Disease, Shanghai Medical Center of Kidney, Shanghai Institute of Kidney and Dialysis, Shanghai Key Laboratory of Kidney and Blood Purification, Hemodialysis Quality Control Center of Shanghai, Shanghai, China; 2 School of Computer Science & Information Engineering, Shanghai Institute of Technology, Shanghai, China

**Keywords:** Chronic kidney disease, Machine learning, Model interpretation, Clinical decision support

## Abstract

**Background:**

Chronic kidney disease (CKD) is a global public health concern. Therefore, to provide timely intervention for non-hospitalized high-risk patients and rationally allocate limited clinical resources is important to mine the key factors when designing a CKD prediction model.

**Methods:**

This study included data from 1,358 patients with CKD pathologically confirmed during the period from December 2017 to September 2020 at Zhongshan Hospital. A CKD prediction interpretation framework based on machine learning was proposed. From among 100 variables, 17 were selected for the model construction through a recursive feature elimination with logistic regression feature screening. Several machine learning classifiers, including extreme gradient boosting, gaussian-based naive bayes, a neural network, ridge regression, and linear model logistic regression (LR), were trained, and an ensemble model was developed to predict 24-hour urine protein. The detailed relationship between the risk of CKD progression and these predictors was determined using a global interpretation. A patient-specific analysis was conducted using a local interpretation.

**Results:**

The results showed that LR achieved the best performance, with an area under the curve (AUC) of 0.850 in a single machine learning model. The ensemble model constructed using the voting integration method further improved the AUC to 0.856. The major predictors of moderate-to-severe severity included lower levels of 25-OH-vitamin, albumin, transferrin in males, and higher levels of cystatin C.

**Conclusions:**

Compared with the clinical single kidney function evaluation indicators (eGFR, Scr), the machine learning model proposed in this study improved the prediction accuracy of CKD progression by 17.6% and 24.6%, respectively, and the AUC was improved by 0.250 and 0.236, respectively. Our framework can achieve a good predictive interpretation and provide effective clinical decision support.

**Supplementary Information:**

The online version contains supplementary material available at 10.1186/s12911-023-02269-2.

## Background

Chronic kidney disease (CKD) affects 5–10% of the global population and is the leading cause of catastrophic health expenditure. It has therefore become a major global public health problem [1]. Furthermore, CKD is projected to become the fifth leading cause of death worldwide by 2040. The compensatory effects of the kidneys make the monitoring of CKD difficult [2]. Clinicians have made significant efforts to determine the key factors that can delay the progression of CKD [3]. Therefore, a risk prediction model for monitoring such progression would be an economical and effective tool [4–6].

With the loss of renal function in CKD patients, the interval between follow-ups recommended by nephrologists becomes shorter, which makes the 24-h urine protein test a heavier medical burden [7]. This burden can be effectively reduced through follow-ups and less time-consuming inspections. Patients with a 24-h urinary protein content less than 1 g/24 h are classified as low-risk, and outpatient follow-up is considered the main treatment. Patients with a 24-h urinary protein content higher than 1 g/24 h are classified as high-risk and assigned to centralized in-hospital management. However, the 24-h quantitative urine protein detection process is complex, involving a lengthy measurement cycle, high patient-compliance requirement, and numerous influencing factors. We are therefore committed to the development of a simple and rapid method to replace the traditional approach.

Compared with traditional scale-based scoring, machine learning (ML) models are widely used in interdisciplinary fields owing to its efficiency, accuracy, and reproducibility. Moreover, it demonstrates significant potential for disease prediction [8]. In comparison to six other machine learning models, Lee et al. achieved an excellent performance when applying a gradient boosting model to malaria prediction [9]. The results of Huang et al. showed that random forest can effectively predict stroke incidence in adult patients with hypertension [10]. The application of machine learning in the field of kidney disease has long been a topic of interest. Various functional methods have been developed for purposes such as predicting the survival rate of dialysis patients [11] and early screening of CKD [12]. Although considerable progress has been made, achieving a good predictability and interpretability remains a considerable challenge. Existing risk prediction models primarily focus on identifying risk factors, and further investigations into the detailed relationship between high-risk factors and CKD risk have rarely been reported.

In current medical studies, new prognostic indicators and their clinical interpretation have received an increasing amount of attention. The screening of such potential clinical indicators has become an important problem. Therefore, several novel feature reduction algorithms have been proposed, including a novel feature reduction (NFR) model [13], an advanced hybrid ensemble gain ratio feature selection (AHEGFS) model [14], and a bio-inspired ensemble feature selection (BEFS) model [15]. Meanwhile, the Shapley additive explanations (SHAP) algorithm has also made exciting discoveries in the use of interpretable techniques in the medical field. SHAP is a method introduced by Lundberg and Lee in 2017 for explaining the predictions of ML models using SHAP values. The key idea of SHAP is to compute SHAP values for each feature of the sample to be explained, to estimate the total effect, main effects, and interaction effects of the variables [16]. Zhao et al. first identified mechanical ventilation and pressure support ventilation as the most important predictive features of extubation failure in intensive care units based on SHAP values [17]. Tseng et al. used SHAP technology to identify important risk factors in acute kidney injury that were ignored by traditional risk scoring models, including intraoperative urine output, IV fluid infusion, blood product transfusion, and dynamic changes in hemodynamics [18]. SHAP interpreters are used to provide a personalized assessment and interpretation of models from both global and local perspectives, ensuring the reliability of prediction results and providing more evidence for solving clinical problems.

Herein, we describe a study conducted with patients having CKD and report a method for CKD prediction and interpretation. Specifically, recursive feature elimination with logistic regression (RFE-LR) was used to identify the risk factors for the progression of kidney disease. Second, based on the random forest (RF) algorithm and voting integration method combined with logistic regression, a risk stratification system for CKD was developed. Finally, the SHAP method was used to explain the prediction model used to support clinical practice and ensure the reliability of the results.

## Materials and methods

**Fig. 1:**
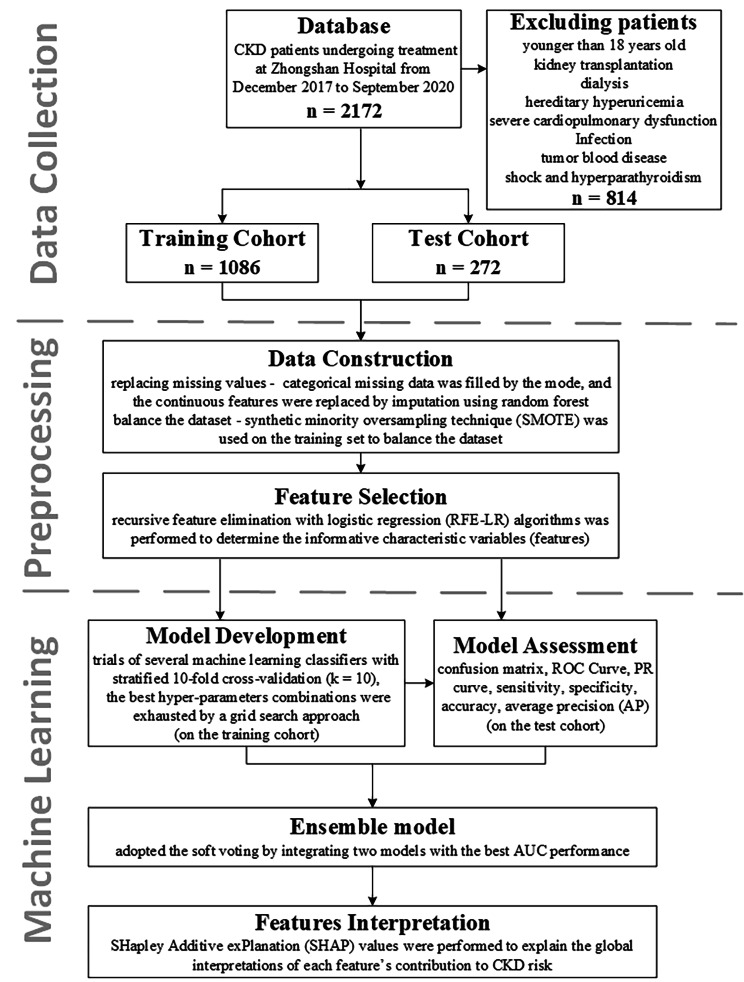
Chronic kidney disease (CKD) prediction and decision support framework. A total of 1,358 patients were included in this study, with 100 clinical variables applied. The data were divided into training (80%) and validation (20%) sets. The model was trained using k-fold cross-validation (k = 10), and a grid search was conducted to determine the best parameter combinations

The study protocol (Fig. 1) received ethical approval from the Ethics Committee of Zhongshan Hospital. The study was conducted in compliance with the World Medical Association Declaration of Helsinki on Ethical Principles for Medical Research Involving Human Subjects, and the national research regulations. Considering the retrospective nature of this study, informed consent was waived by the Ethics Committee of Zhongshan Hospital.

### Study participants

From the database, we retrospectively selected 1,358 patients with pathologically confirmed CKD from December 2017 to September 2020. Patients younger than 18 years and those who underwent kidney transplantation or dialysis or had a diagnosis of hereditary hyperuricemia, severe cardiopulmonary dysfunction, infection, tumor blood disease, shock, or hyperparathyroidism were excluded from all analyses.

We collected treatment data and then retrospectively extracted the clinical characteristics, such as demographics, routine blood tests, blood biochemistry, and blood immunity of the patients from electronic medical records and entered them into our structured database.

### Study outcome

In our study, the prediction targets are represented in binary form (0 = negative, 1 = positive). The outcome of the present study was the status of 24-h urinary protein, which was judged based on whether the urine protein level was lower or higher than 1 g/24 h, defined as mild (negative) or advanced (positive), respectively.

### Data construction and feature selection

We collected 100 easily obtainable clinical features from our database. The proportion of missing values for all features was < 10%. Missing categorical data were filled in based on the mode, and continuous features were replaced through an imputation using RF [19]. The categorical features were then transformed into binary dummy variables. The dataset was randomly divided into a training cohort (80%) and an independent test cohort (20%), and synthetic minority oversampling technique (SMOTE) was used on the training set to balance the dataset. To identify whether any subsets of the features can achieve a better discrimination than the initial set of features and to determine the informative characteristic variables (features) in the prediction of CKD, the RFE-LR and least absolute shrinkage and selection operator (LASSO) algorithms were used.

### Model development and assessment

For the development system, in this study, we used macOS Monterey (Apple M1 Pro) with 16 GB of memory. As the analysis software, Python version v3.10 and the sklearn v1.1.1 machine learning library were utilized as the main analysis tool. The model development included trials using several different machine learning classifiers, such as extreme gradient boosting (XGBoost) models [20], gaussian-based naive bayes [21], a neural network (NN) [22], ridge regression [23], and linear model logistic regression (LR) [24]. A brief description of these algorithms is described in the model establishment and brief illustrations (Additional file 1). We trained the models using a stratified k-fold cross-validation (k = 10) applied to the training cohort, and determined the best hyperparameter combinations through a grid search approach.

To quantify the discriminative capabilities of the model, we plotted the receiver operating characteristic (ROC) and precision–recall curves based on a confusion matrix, and then calculated the area under the ROC curve (AUC), which was used as the main metric to assess the model performance. Furthermore, the sensitivity, specificity, accuracy, average precision, and execution time were used to evaluate the model performance from multiple perspectives. The calculation principles of these assessment indicators are described in the performance metrics section (Additional file 1). In addition, we adopted a soft voting ensemble model by integrating the two models with the best AUC.

### Feature interpretation

Feature importance refers to the extent to which the elimination of feature information increases the model error, which provides a highly compressed global insight into the behavior of the model. We computed the SHAP values to evaluate the correctness of the feature interpretation in the best-performing model and explain the global interpretations of each feature contribution to the risk of CKD.

## Results

### Patients and clinical characteristics

In the final cohort, we reviewed the medical records of 1,358 patients with CKD who underwent treatment at Zhongshan Hospital from December 2017 to September 2020. The mean age was 51.12 ± 16.09 years, and 910 (67.01%) of the patients were male. A total of 906 (68%) and 452 (32%) subjects were classified as patients having advanced (positive) and mild (negative) CKD, respectively. In addition, after applying data balance processing of the SMOTE algorithm on the training set, 364 negative samples were oversampled, and thus the sample ratio of the final training dataset was 1 (both at 725). The estimated glomerular filtration rate (eGFR) was calculated using the MDRD formula. The proportions of missing values of the included clinical features were all < 10%. After data preprocessing, 100 complete clinical variables were used as predictive variables, the baseline characteristics of which are shown in Table 1.

**Table 1:** Baseline characteristics of included CKD patients

	Total (N = 1358)	Mild CKD (N = 452)	Advanced CKD (N = 906)	P value
**Gender**				
Female	448 (32.99%)	182 (40.27%)	266 (29.36%)	< 0.01
Male	910 (67.01%)	270 (59.73%)	640 (70.64%)	
**Age**				
Mean (SD)	51.12 ± 16.09	49.56 ± 16.33	51.90 ± 15.92	0.01
**DBIL**				
Mean (SD)	2.11 ± 3.03	2.50 ± 1.71	1.92 ± 3.48	< 0.01
Missing	133 (9.8%)	51 (11.3%)	82 (9.1%)	
**TP**				
Mean (SD)	59.96 ± 10.23	65.50 ± 6.56	57.26 ± 10.61	< 0.01
Missing	131 (9.6%)	50 (11.1%)	81 (8.9%)	
**ALB**				
Mean (SD)	35.09 ± 7.71	39.67 ± 4.74	32.86 ± 7.90	< 0.01
Missing	133 (9.8%)	51 (11.3%)	82 (9.1%)	
**GLO**				
Mean (SD)	24.87 ± 4.98	25.85 ± 4.31	24.40 ± 5.21	< 0.01
Missing	133 (9.8%)	51 (11.3%)	82 (9.1%)	
**AGRatio**				
Mean (SD)	1.44 ± 0.35	1.57 ± 0.30	1.38 ± 0.35	< 0.01
Missing	133 (9.8%)	51 (11.3%)	82 (9.1%)	
**SPE.ALB**				
Mean (SD)	55.75 ± 6.01	58.15 ± 4.66	54.56 ± 6.26	< 0.01
Missing	164 (12.1%)	55 (12.2%)	109 (12.0%)	
**SPE.alpha1**				
Mean (SD)	4.61 ± 1.32	4.08 ± 1.07	4.87 ± 1.36	< 0.01
Missing	164 (12.1%)	55 (12.2%)	109 (12.0%)	
**SPE.alpha2**				
Mean (SD)	11.55 ± 4.25	9.51 ± 2.31	12.57 ± 4.62	< 0.01
Missing	164 (12.1%)	55 (12.2%)	109 (12.0%)	
**SPE.beta**				
Mean (SD)	12.22 ± 1.85	11.66 ± 1.45	12.50 ± 1.96	< 0.01
Missing	164 (12.1%)	55 (12.2%)	109 (12.0%)	
**SPE.gamma**				
Mean (SD)	15.88 ± 4.34	16.60 ± 3.62	15.52 ± 4.61	< 0.01
Missing	164 (12.1%)	55 (12.2%)	109 (12.0%)	
**ALT**				
Mean (SD)	20.62 ± 23.20	21.87 ± 29.67	20.01 ± 19.29	0.25
Missing	125 (9.2%)	50 (11.1%)	75 (8.3%)	
**AST**				
Mean (SD)	19.66 ± 14.29	19.92 ± 19.20	19.53 ± 11.16	0.70
Missing	133 (9.8%)	51 (11.3%)	82 (9.1%)	
**ALP**				
Mean (SD)	68.64 ± 32.29	67.58 ± 27.48	69.15 ± 34.39	0.39
Missing	133 (9.8%)	51 (11.3%)	82 (9.1%)	
**GGT**				
Mean (SD)	35.48 ± 59.54	35.77 ± 78.09	35.35 ± 48.12	0.92
Missing	125 (9.2%)	50 (11.1%)	75 (8.3%)	
**TBA**				
Mean (SD)	4.65 ± 5.69	5.57 ± 7.35	4.19 ± 4.58	< 0.01
Missing	164 (12.1%)	55 (12.2%)	109 (12.0%)	
**LDH**				
Mean (SD)	196.88 ± 55.63	181.08 ± 47.35	204.76 ± 57.77	< 0.01
Missing	165 (12.2%)	55 (12.2%)	110 (12.1%)	
**APLB1**				
Mean (SD)	127.40 ± 144.43	118.27 ± 141.11	131.95 ± 145.93	0.12
Missing	164 (12.1%)	55 (12.2%)	109 (12.0%)	
**BUN**				
Mean (SD)	10.88 ± 7.78	8.84 ± 6.55	11.88 ± 8.13	< 0.01
Missing	74 (5.4%)	32 (7.1%)	42 (4.6%)	
**CRE**				
Mean (SD)	205.18 ± 198.66	158.54 ± 152.71	227.85 ± 213.95	< 0.01
Missing	74 (5.4%)	32 (7.1%)	42 (4.6%)	
**eGFR**				
Mean (SD)	54.21 ± 33.96	62.20 ± 32.34	50.33 ± 34.07	< 0.01
Missing	74 (5.4%)	32 (7.1%)	42 (4.6%)	
**CYSC**				
Mean (SD)	1.94 ± 1.24	1.64 ± 1.09	2.09 ± 1.28	< 0.01
Missing	362 (26.7%)	119 (26.3%)	243 (26.8%)	
**UA**				
Mean (SD)	399.96 ± 107.38	376.30 ± 103.29	411.46 ± 107.51	< 0.01
Missing	74 (5.4%)	32 (7.1%)	42 (4.6%)	
**GA**				
Mean (SD)	12.82 ± 3.68	13.86 ± 3.25	12.32 ± 3.78	< 0.01
Missing	241 (17.7%)	88 (19.5%)	153 (16.9%)	
**TG**				
Mean (SD)	2.05 ± 1.52	1.81 ± 1.19	2.16 ± 1.65	< 0.01
Missing	201 (14.8%)	68 (15.0%)	133 (14.7%)	
**LDL**				
Mean (SD)	2.82 ± 1.49	2.40 ± 0.99	3.03 ± 1.64	< 0.01
Missing	201 (14.8%)	68 (15.0%)	133 (14.7%)	
**N.HDL**				
Mean (SD)	3.71 ± 1.63	3.20 ± 1.09	3.97 ± 1.79	< 0.01
Missing	202 (14.9%)	68 (15.0%)	134 (14.8%)	
**APO.A.I**				
Mean (SD)	1.33 ± 0.38	1.27 ± 0.30	1.36 ± 0.42	< 0.01
Missing	202 (14.9%)	68 (15.0%)	134 (14.8%)	
**APO.B**				
Mean (SD)	0.93 ± 0.36	0.82 ± 0.26	0.98 ± 0.39	< 0.01
Missing	202 (14.9%)	68 (15.0%)	134 (14.8%)	
**APO.E**				
Mean (SD)	52.10 ± 25.55	46.03 ± 19.34	55.11 ± 27.65	< 0.01
Missing	202 (14.9%)	68 (15.0%)	134 (14.8%)	
**LPA1**				
Mean (SD)	229.06 ± 328.97	199.44 ± 300.57	243.79 ± 341.44	0.02
Missing	202 (14.9%)	68 (15.0%)	134 (14.8%)	
**Na**				
Mean (SD)	141.68 ± 2.63	141.76 ± 2.50	141.64 ± 2.69	0.43
Missing	80 (5.9%)	35 (7.7%)	45 (5.0%)	
**K**				
Mean (SD)	4.10 ± 0.48	4.07 ± 0.42	4.11 ± 0.51	0.13
Missing	80 (5.9%)	35 (7.7%)	45 (5.0%)	
**Cl**				
Mean (SD)	105.13 ± 3.62	104.57 ± 3.25	105.39 ± 3.77	< 0.01
Missing	80 (5.9%)	35 (7.7%)	45 (5.0%)	
**CO2**				
Mean (SD)	25.34 ± 3.29	25.47 ± 3.03	25.27 ± 3.41	0.28
Missing	80 (5.9%)	35 (7.7%)	45 (5.0%)	
**AG**				
Mean (SD)	11.21 ± 2.76	11.71 ± 2.39	10.97 ± 2.89	< 0.01
Missing	80 (5.9%)	35 (7.7%)	45 (5.0%)	
**Ca**				
Mean (SD)	2.17 ± 0.20	2.25 ± 0.18	2.14 ± 0.19	< 0.01
Missing	95 (7.0%)	42 (9.3%)	53 (5.8%)	
**P**				
Mean (SD)	1.26 ± 0.30	1.21 ± 0.26	1.28 ± 0.31	< 0.01
Missing	96 (7.1%)	43 (9.5%)	53 (5.8%)	
**Mg**				
Mean (SD)	0.85 ± 0.10	0.86 ± 0.09	0.85 ± 0.10	0.01
Missing	96 (7.1%)	43 (9.5%)	53 (5.8%)	
**CPK**				
Mean (SD)	108.01 ± 125.32	89.95 ± 74.79	116.94 ± 143.08	< 0.01
Missing	188 (13.8%)	65 (14.4%)	123 (13.6%)	
**CK.MB**				
Mean (SD)	15.59 ± 11.07	14.51 ± 11.25	16.13 ± 10.95	0.02
Missing	186 (13.7%)	63 (13.9%)	123 (13.6%)	
**CK.MM**				
Mean (SD)	95.45 ± 156.53	84.19 ± 183.31	101.05 ± 141.16	0.11
Missing	186 (13.7%)	63 (13.9%)	123 (13.6%)	
**CRP**				
Mean (SD)	5.86 ± 16.71	6.02 ± 17.04	5.78 ± 16.56	0.82
Missing	144 (10.6%)	55 (12.2%)	89 (9.8%)	
**HCY**				
Mean (SD)	16.89 ± 12.92	15.99 ± 9.23	17.34 ± 14.39	0.06
Missing	266 (19.6%)	90 (19.9%)	176 (19.4%)	
**IRON**				
Mean (SD)	14.28 ± 6.01	15.09 ± 5.82	13.88 ± 6.06	< 0.01
Missing	222 (16.3%)	81 (17.9%)	141 (15.6%)	
**UIBC**				
Mean (SD)	29.90 ± 10.74	33.78 ± 9.58	28.02 ± 10.77	< 0.01
Missing	223 (16.4%)	82 (18.1%)	141 (15.6%)	
**TIBC**				
Mean (SD)	44.22 ± 10.56	48.94 ± 8.59	41.94 ± 10.67	< 0.01
Missing	223 (16.4%)	82 (18.1%)	141 (15.6%)	
**TS.**				
Mean (SD)	33.49 ± 14.67	31.55 ± 13.05	34.43 ± 15.31	< 0.01
Missing	223 (16.4%)	82 (18.1%)	141 (15.6%)	
**IGG**				
Mean (SD)	9.79 ± 4.10	11.20 ± 3.40	9.11 ± 4.24	< 0.01
Missing	206 (15.2%)	76 (16.8%)	130 (14.3%)	
**IGA**				
Mean (SD)	2.48 ± 1.10	2.56 ± 1.13	2.44 ± 1.09	0.09
Missing	234 (17.2%)	84 (18.6%)	150 (16.6%)	
**RBP**				
Mean (SD)	33.06 ± 38.51	28.30 ± 34.27	35.47 ± 40.30	< 0.01
Missing	358 (26.4%)	116 (25.7%)	242 (26.7%)	
**IGM**				
Mean (SD)	0.96 ± 0.51	0.98 ± 0.56	0.95 ± 0.48	0.32
Missing	234 (17.2%)	84 (18.6%)	150 (16.6%)	
**IGE**				
Mean (SD)	191.62 ± 702.02	136.94 ± 356.09	218.39 ± 818.68	0.02
Missing	217 (16.0%)	77 (17.0%)	140 (15.5%)	
**C3**				
Mean (SD)	0.97 ± 0.20	0.96 ± 0.18	0.97 ± 0.21	0.34
Missing	228 (16.8%)	80 (17.7%)	148 (16.3%)	
**C4**				
Mean (SD)	0.23 ± 0.07	0.22 ± 0.06	0.23 ± 0.07	< 0.01
Missing	228 (16.8%)	80 (17.7%)	148 (16.3%)	
**CH50**				
Mean (SD)	56.26 ± 19.92	56.29 ± 19.08	56.25 ± 20.33	0.97
Missing	227 (16.7%)	79 (17.5%)	148 (16.3%)	
**B2M**				
Mean (SD)	5.50 ± 4.95	4.49 ± 4.80	6.00 ± 4.94	< 0.01
Missing	324 (23.9%)	106 (23.5%)	218 (24.1%)	
**TRF**				
Mean (SD)	1.90 ± 0.46	2.11 ± 0.39	1.80 ± 0.46	< 0.01
Missing	234 (17.2%)	85 (18.8%)	149 (16.4%)	
**ASO**				
Mean (SD)	62.70 ± 93.47	68.73 ± 81.80	59.72 ± 98.65	0.12
Missing	320 (23.6%)	109 (24.1%)	211 (23.3%)	
**RF**				
Mean (SD)	11.54 ± 27.87	12.39 ± 39.63	11.13 ± 19.59	0.58
Missing	321 (23.6%)	108 (23.9%)	213 (23.5%)	
**KAP**				
Mean (SD)	2.69 ± 1.11	3.00 ± 0.95	2.53 ± 1.16	< 0.01
Missing	331 (24.4%)	109 (24.1%)	222 (24.5%)	
**LAM**				
Mean (SD)	1.57 ± 0.65	1.76 ± 0.61	1.47 ± 0.65	< 0.01
Missing	331 (24.4%)	109 (24.1%)	222 (24.5%)	
**CA199**				
Mean (SD)	17.37 ± 52.51	13.63 ± 26.38	19.11 ± 60.89	0.05
Missing	374 (27.5%)	140 (31.0%)	234 (25.8%)	
**NSE**				
Mean (SD)	13.17 ± 4.04	12.79 ± 3.71	13.34 ± 4.17	0.04
Missing	385 (28.4%)	143 (31.6%)	242 (26.7%)	
**T3**				
Mean (SD)	1.45 ± 0.38	1.54 ± 0.35	1.42 ± 0.39	< 0.01
Missing	343 (25.3%)	125 (27.7%)	218 (24.1%)	
**T4**				
Mean (SD)	84.29 ± 19.58	89.40 ± 18.27	81.86 ± 19.72	< 0.01
Missing	342 (25.2%)	125 (27.7%)	217 (24.0%)	
**FT3**				
Mean (SD)	4.12 ± 1.01	4.44 ± 0.93	3.97 ± 1.01	< 0.01
Missing	339 (25.0%)	122 (27.0%)	217 (24.0%)	
**FT4**				
Mean (SD)	15.14 ± 2.90	15.97 ± 2.74	14.74 ± 2.89	< 0.01
Missing	339 (25.0%)	122 (27.0%)	217 (24.0%)	
**TSH**				
Mean (SD)	3.81 ± 7.37	3.54 ± 7.55	3.94 ± 7.29	0.43
Missing	339 (25.0%)	123 (27.2%)	216 (23.8%)	
**PTH**				
Mean (SD)	75.98 ± 103.56	63.57 ± 74.62	82.27 ± 115.02	< 0.01
Missing	204 (15.0%)	64 (14.2%)	140 (15.5%)	
**TDB**				
Mean (SD)	314.84 ± 277.94	282.47 ± 278.80	330.78 ± 276.32	< 0.01
Missing	224 (16.5%)	78 (17.3%)	146 (16.1%)	
**B12**				
Mean (SD)	525.56 ± 309.05	516.47 ± 293.20	530.01 ± 316.61	0.48
Missing	229 (16.9%)	81 (17.9%)	148 (16.3%)	
**FOL**				
Mean (SD)	8.54 ± 5.08	8.85 ± 4.94	8.39 ± 5.14	0.14
Missing	230 (16.9%)	81 (17.9%)	149 (16.4%)	
**NTX**				
Mean (SD)	32.78 ± 41.57	27.02 ± 30.97	35.64 ± 45.68	< 0.01
Missing	303 (22.3%)	102 (22.6%)	201 (22.2%)	
**X25OHD**				
Mean (SD)	31.84 ± 19.43	42.14 ± 21.53	26.59 ± 15.89	< 0.01
Missing	216 (15.9%)	66 (14.6%)	150 (16.6%)	
**HGB**				
Mean (SD)	119.29 ± 25.81	123.62 ± 23.45	117.16 ± 26.65	< 0.01
Missing	90 (6.6%)	34 (7.5%)	56 (6.2%)	
**HCT**				
Mean (SD)	35.65 ± 7.40	36.91 ± 6.57	35.03 ± 7.71	< 0.01
Missing	90 (6.6%)	34 (7.5%)	56 (6.2%)	
**MCV**				
Mean (SD)	90.05 ± 5.06	90.05 ± 5.28	90.05 ± 4.95	0.99
Missing	90 (6.6%)	34 (7.5%)	56 (6.2%)	
**MCH**				
Mean (SD)	30.10 ± 1.98	30.11 ± 1.91	30.10 ± 2.01	0.90
Missing	90 (6.6%)	34 (7.5%)	56 (6.2%)	
**MCHC**				
Mean (SD)	334.24 ± 12.17	334.45 ± 11.69	334.14 ± 12.40	0.66
Missing	90 (6.6%)	34 (7.5%)	56 (6.2%)	
**PLT**				
Mean (SD)	218.90 ± 67.67	217.56 ± 64.12	219.56 ± 69.37	0.61
Missing	102 (7.5%)	38 (8.4%)	64 (7.1%)	
**WBC**				
Mean (SD)	30.92 ± 496.32	54.03 ± 853.94	19.37 ± 70.94	0.39
Missing	20 (1.5%)	6 (1.3%)	14 (1.5%)	
**NEUT.**				
Mean (SD)	61.92 ± 11.70	59.65 ± 11.73	63.03 ± 11.53	< 0.01
Missing	90 (6.6%)	34 (7.5%)	56 (6.2%)	
**LYMPH.**				
Mean (SD)	27.00 ± 10.00	29.16 ± 9.96	25.94 ± 9.85	< 0.01
Missing	90 (6.6%)	34 (7.5%)	56 (6.2%)	
**MONO.**				
Mean (SD)	7.78 ± 2.34	7.89 ± 2.30	7.72 ± 2.36	0.24
Missing	90 (6.6%)	34 (7.5%)	56 (6.2%)	
**EO.**				
Mean (SD)	2.86 ± 2.77	2.83 ± 2.57	2.88 ± 2.87	0.80
Missing	90 (6.6%)	34 (7.5%)	56 (6.2%)	
**BASO.**				
Mean (SD)	0.44 ± 0.27	0.47 ± 0.27	0.42 ± 0.26	< 0.01
Missing	90 (6.6%)	34 (7.5%)	56 (6.2%)	
**NEUT**				
Mean (SD)	4.47 ± 2.42	4.13 ± 2.03	4.64 ± 2.58	< 0.01
Missing	90 (6.6%)	34 (7.5%)	56 (6.2%)	
**LYMPH**				
Mean (SD)	376.29 ± 780.47	368.01 ± 811.23	380.34 ± 765.38	0.79
Missing	79 (5.8%)	31 (6.9%)	48 (5.3%)	
**MONO**				
Mean (SD)	0.52 ± 0.20	0.51 ± 0.18	0.53 ± 0.21	0.15
Missing	90 (6.6%)	34 (7.5%)	56 (6.2%)	
**EO**				
Mean (SD)	0.18 ± 0.19	0.18 ± 0.18	0.18 ± 0.20	0.56
Missing	90 (6.6%)	34 (7.5%)	56 (6.2%)	
**BASO**				
Mean (SD)	0.03 ± 0.02	0.03 ± 0.02	0.03 ± 0.02	0.17
Missing	90 (6.6%)	34 (7.5%)	56 (6.2%)	
**RDW.CV**				
Mean (SD)	12.99 ± 1.35	12.86 ± 1.34	13.06 ± 1.35	0.01
Missing	102 (7.5%)	38 (8.4%)	64 (7.1%)	
**RDW.SD**				
Mean (SD)	42.46 ± 4.71	42.05 ± 4.90	42.66 ± 4.61	0.03
Missing	102 (7.5%)	38 (8.4%)	64 (7.1%)	
**MPV**				
Mean (SD)	10.95 ± 1.10	11.00 ± 1.05	10.93 ± 1.12	0.29
Missing	104 (7.7%)	37 (8.2%)	67 (7.4%)	
**PCT**				
Mean (SD)	0.24 ± 0.06	0.24 ± 0.06	0.24 ± 0.07	0.74
Missing	104 (7.7%)	37 (8.2%)	67 (7.4%)	
**P.LCR**				
Mean (SD)	32.44 ± 8.97	32.89 ± 8.59	32.21 ± 9.16	0.19
Missing	104 (7.7%)	37 (8.2%)	67 (7.4%)	
**PDW**				
Mean (SD)	13.08 ± 2.57	13.23 ± 2.36	13.01 ± 2.66	0.14
Missing	116 (8.5%)	41 (9.1%)	75 (8.3%)	
**RET.**				
Mean (SD)	1.67 ± 0.95	1.64 ± 0.96	1.68 ± 0.94	0.50
Missing	228 (16.8%)	76 (16.8%)	152 (16.8%)	
**U.PRO**				
Mean (SD)	2.70 ± 3.02	0.52 ± 0.32	3.79 ± 3.17	< 0.01

### Feature selection

**Fig. 2:**
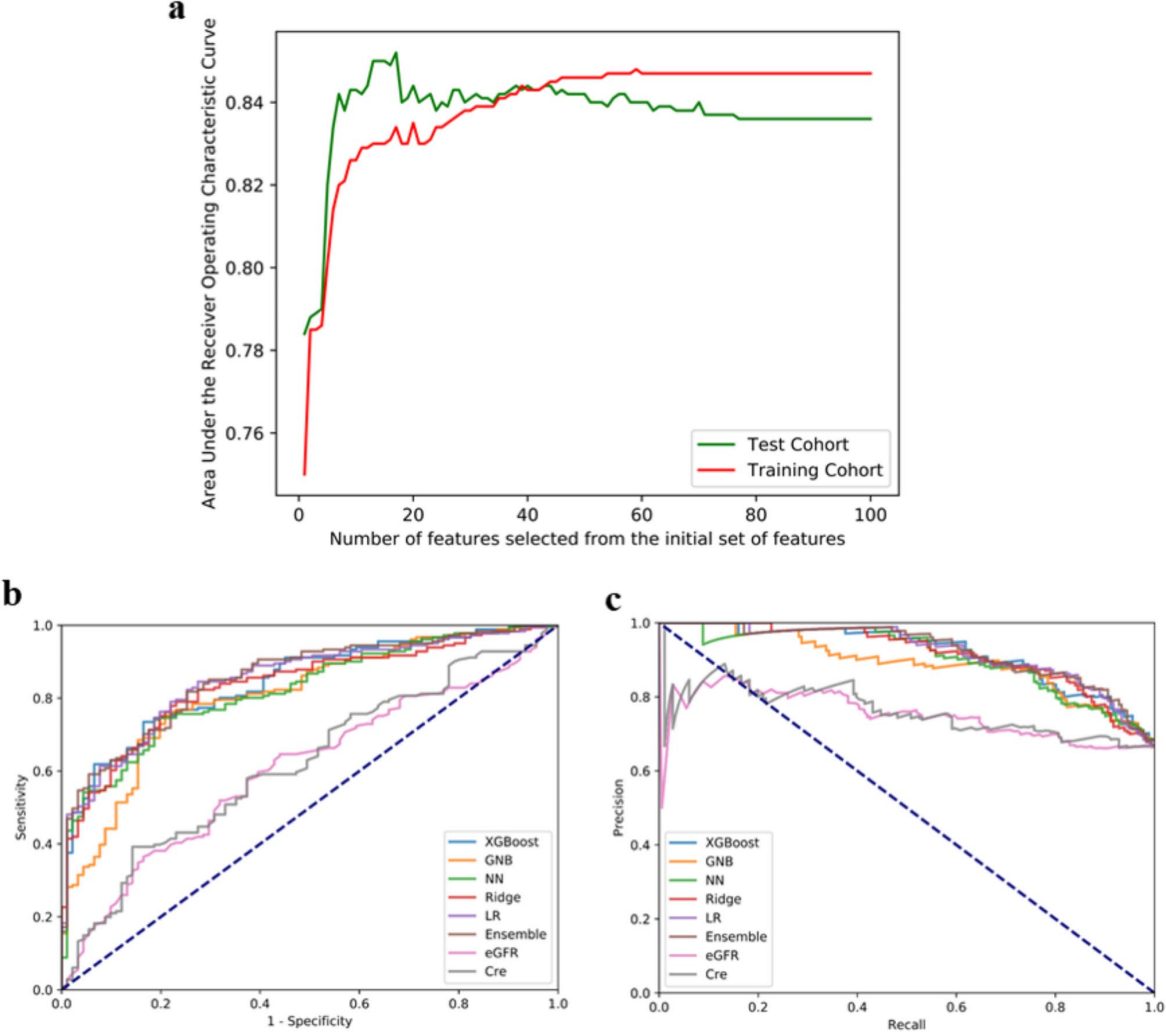
Screening of predictors and evaluation of models. (a) RFE-LR used to examine whether any subset of the input features can achieve a better discrimination than the initial set of features. (b) ROC curves of different models on the validation sets. (c) Precision–recall (PR) curves of different models on the validation sets

After imputation, we compared the results of the model construction without feature screening and with RFE-LR and LASSO feature screening, and then used the AUC as the main evaluation index of the model. In the model construction results without feature screening, the highest AUC was 0.833 (Table S3). A total of 21 feature indexes were obtained through LASSO feature screening based on the optimal penalty parameter λ (0.035) using the 1 − standard error (SE) criterion (Figure S1), which achieved the highest AUC of 0.828 (Table S4). In the results of the RFE-LR feature screening, the performance of the model was significantly improved when 17 variables were used (Fig. 2a), and the model showed an over-fitting with a further increase in the number of variables. The highest AUC was 0.85 after RFE-LR feature screening (Table 2). In brief, the RFE-LR algorithm was used to reduce the number of feature variables to 17, which achieved the highest accuracy and AUC compared to using all features separately, with an improvement of 3.3% and 0.017, respectively. Based on the results of the AUC comparison, we conducted a follow-up study using the results of RFE-LR. We then used these 17 variables for subsequent model building, including gender, total protein (TP), albumin (ALB), serum protein electrophoresis-albumin (SPE-ALB), serum protein electrophoresis-alpha2 (SPE-alpha2), serum protein electrophoresis-beta (SPE-beta), eGFR, cystatin C (CYSC), uric acid (UA), glycated albumin (GA), non high density lipoprotein (N-HDL), apolipoprotein A (APO-A-I), creatine phosphokinase (CPK), retinol conjugated protein (RBP), transferrin (TRF), lambda light chain (LAM), and 25 Hydroxyvitamin D (25OHD).

**Table 2:** Results of hyper-parameter optimization of different machine learning algorithms

Model	Hyper-parameter space	Best Combination of Hyperparameters	AUC in the training cohort	AUC in the test cohort
XGBoost	{‘max_depth’: [2, 3, 5–7, 9, 12, 15, 17, 25], ‘min_child_weight’: [1, 3, 5, 7], ‘gamma’:[ 0, 0.05 ,0.1,0.2, 0.3, 0.5, 0.7, 0.9, 1], ‘subsample’:[ 0.6, 0.7, 0.8, 0.9, 1], ‘colsample_bytree’:[0.6, 0.7, 0.8, 0.9, 1], ‘learning_rate’:[0.01, 0.015, 0.025, 0.05, 0.1]}	{‘max_depth’: [2], ‘min_child_weight’: [3], ‘gamma’:[0.2], ‘subsample’:[ 0.7], ‘colsample_bytree’:[0.8], ‘learning_rate’:[0.01]}	0.903	0.844
GNB	/	/	0.797	0.808
NN	{‘alpha’: [0.1, 0.01, 0.001, 0.0001], ‘hidden_layer_sizes’:[(50,),(100,)], ‘solver’:[‘sgd’, ‘adam’], ‘activation’:[‘tanh’,‘relu’], ‘learning_rate’:[‘constant’, ‘adaptive’]}	{‘activation’: ‘tanh’, ‘alpha’: 0.1, ‘hidden_layer_sizes’:(50,), ‘learning_rate’: ‘constant’, ‘solver’: ‘adam’}	0.855	0.822
Ridge	{‘alpha’: [0.001, 0.01, 0.1, 1, 10, 100, 1000],‘solver’:[‘svd’, ‘cholesky’, ‘lsqr’, ‘sparse_cg’, ‘sag’, ‘saga’]}	{‘alpha’: 10, ‘solver’: ‘svd’}	0.829	0.836
LR	{‘C’: [0.001, 0.01, 0.1, 1, 10, 100], ‘penalty’:[‘l2’], ‘solver’: [‘newton-cg’, ‘lbfgs’, ‘liblinear’, ‘sag’, ‘saga’]}	{‘C’: 0.1, ‘penalty’: ‘l2’, ‘solver’: ‘newton-cg’}	0.833	0.850

### Model comparison

The adjustment results of the model hyperparameters were summarized firstly. Before adjusting these hyperparameters, the LR model achieved the highest AUC (0.839) (Table S5). Four machine learning models were constructed based on the best hyperparameter combinations of the algorithms (Table 2). The results of the confusion matrixes are summarized in Table 3, where XGBoost created the minimum number of false positives (15) and LR created the maximum number of true positives (153). As can be seen from Table 4; Fig. 2b **and c**, LR achieved the best AUC (0.850) in the single machine learning model. The ensemble model constructed using the voting ensemble method further improved the predictive power and achieved the highest performance (AUC: 0.856). The model with the best sensitivity applied LR (0.845). The specificity values of XGBoost, NN, and the traditional creatinine (Cre) indicator were all above 0.8, whereas the sensitivity of Cre was low (0.392). When compared with the pre-existing single renal function evaluation indices (eGFR, Scr), the prediction performance of machine learning for the progression of CKD was significantly improved (Table 4). In addition, we also compared the running time of different machine learning models under the same hardware conditions. As shown in Table S6, there is little difference among the models in the test cohort. However, when training the cohort of each hyperparameter, GNB had the fastest and XGBoost had the slowest execution time.

**Table 3:** Confusion matrices

Model	Predictive	Actual	
		Mild	Advanced
XGBoost	Mild	76	48
	Advanced	15	133
GNB	Mild	71	43
	Advanced	20	138
NN	Mild	74	47
	Advanced	17	134
Ridge	Mild	70	38
	Advanced	21	143
LR	Mild	66	28
	Advanced	25	153
Ensemble	Mild	68	33
	Advanced	23	148
eGFR	Mild	51	64
	Advanced	40	117
Cre	Mild	78	110
	Advanced	13	71

**Table 4:** Performance summary

Models	AUC	95%CI		sensitivity	specificity	accuracy	AP
		Lower bound	Upper bound				
XGBoost	0.844	0.798	0.891	0.735	0.835	0.768	0.920
GNB	0.808	0.755	0.861	0.762	0.780	0.768	0.893
NN	0.822	0.773	0.872	0.740	0.813	0.765	0.907
Ridge	0.836	0.788	0.884	0.790	0.769	0.783	0.918
LR	0.850	0.805	0.896	0.845	0.725	0.805	0.924
Ensemble	0.856	0.812	0.901	0.818	0.747	0.794	0.926
eGFR	0.606	0.537	0.675	0.646	0.560	0.618	0.753
Cre	0.620	0.551	0.689	0.392	0.857	0.548	0.763

### Most important predictors of CKD risk

**Fig. 3:**
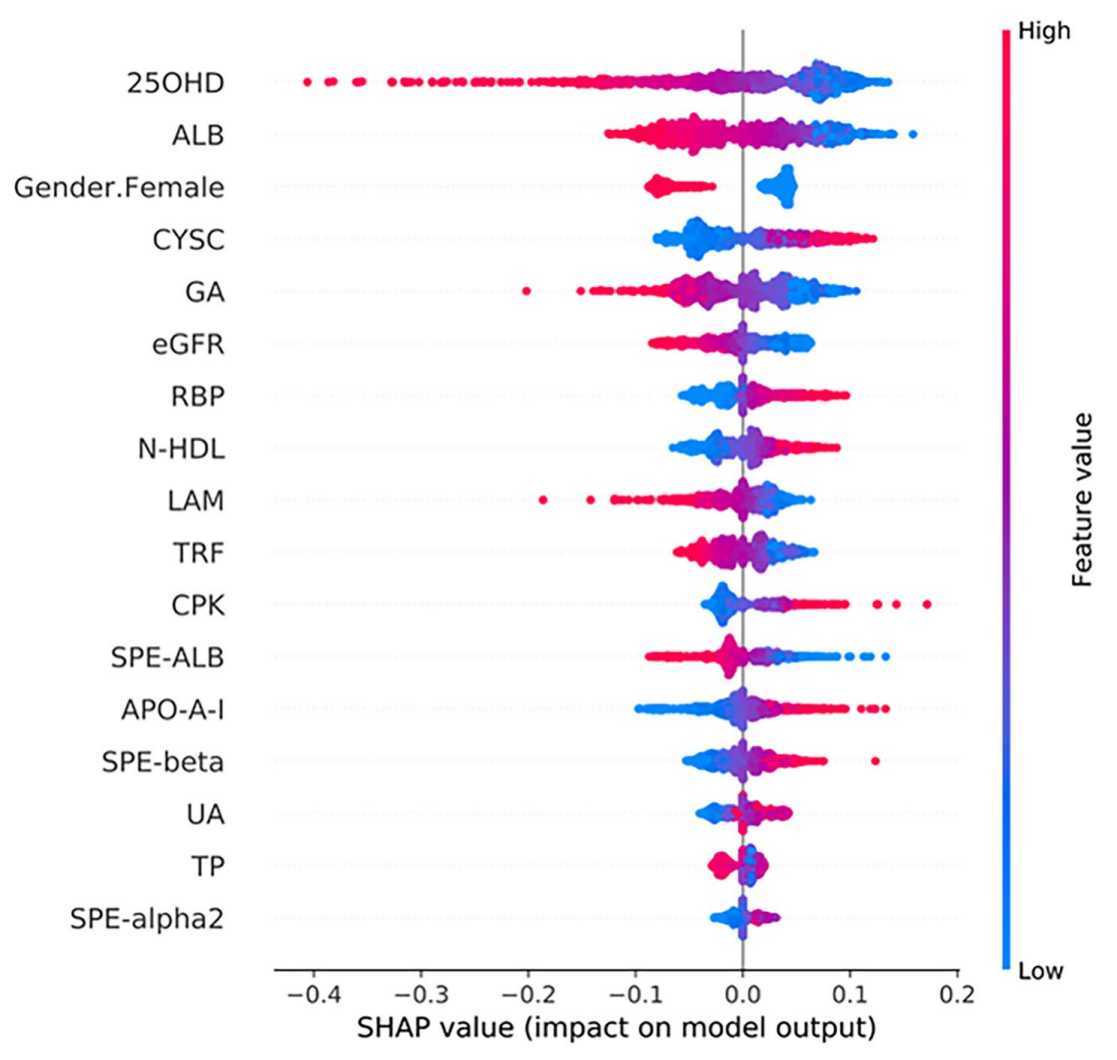
SHAP summary plot of the top-17 features of the ensemble model. The abscissa is the SHAP value, which represents the impact on the model output. The ordinates are different features, with red representing larger eigenvalues, and blue indicating smaller eigenvalues

**Fig. 4:**
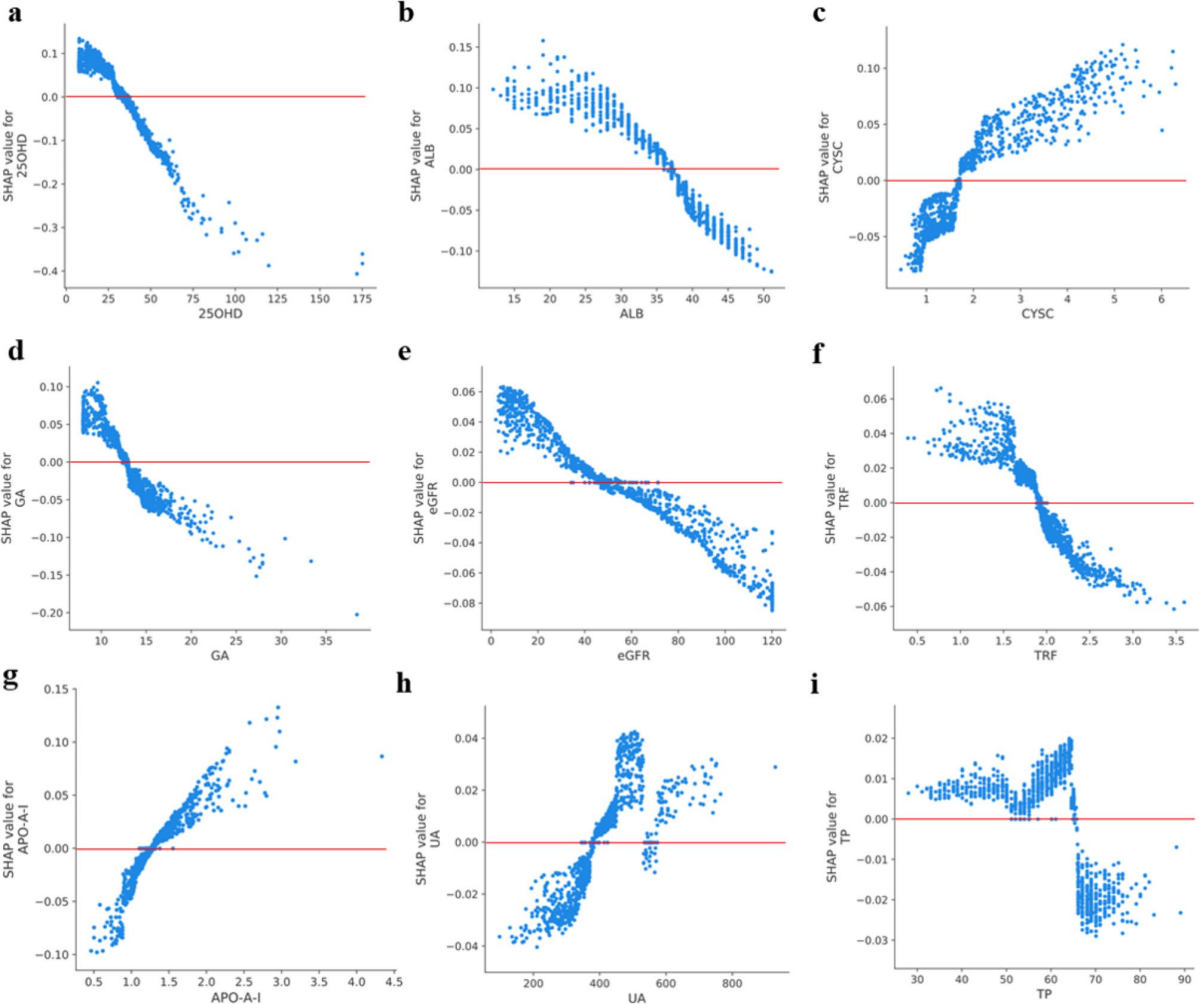
SHAP dependence plots for ensemble model. The SHAP-dependence plot shows the effect of a single feature on the output of the ensemble prediction model. SHAP values for specific features exceeding zero represent an increased risk of CKD progression. (**a-f**) 25-hydroxyvitamin D, albumin, cystatin C, glycated albumin, estimated glomerular filtration rate (eGFR), transferrin, protein A1, uric acid, and total protein

To identify the features influencing the model and their impact on the risk of CKD as a way to support clinical decision-making, a particular variant of SHAP for kernel-based explainers was used for the ensemble model interpretation with the best AUC performance. The features ranked based on the SHAP values in the training dataset are shown in Fig. 3. Features other than Scr and eGFR were discussed to highlight those that may need to be closely monitored. As shown in Fig. 3, lower levels of 25OHD, ALB, and transferrin (TRF), male sex, and higher levels of CYSC were the major predictors of moderate-to-high severity. In addition, to obtain the exact form of the relationship, SHAP-dependence plots (Fig. 4) were employed. A SHAP value exceeding zero is regarded as the cut-off point, and the critical point corresponding to each feature can be observed at this time. According to the results, 25OHD levels lower than 30 nmol/L indicate a moderate or even severe loss of renal function. In addition, when the 25OHD level was higher than 75 nmol/L, the individual differences increased. A decrease in serum ALB level predicts an increase in the risk of CKD. ALB levels below 37 g/L were correlated with a positive predictive value. We also found that the accumulation of CYSC indicates an increased risk of CKD, that is, when the CYSC level is higher than 2 mg/L, the same level of CYSC accounts for a greater difference among the patients. In addition, a higher glycated albumin (GA) level (%) indicates an increased risk of CKD. The results also illustrate the tendency of CKD risk when eGFR levels decrease. An eGFR level below 60 ml/min/1.73 m^2^ is correlated with a positive predictive value. Within the range of 1.5–2.0 g/L, TRF changes slightly, whereas SHAP increases sharply, which shows that attention should be paid to changes in the TRF. Such analyses can help clinicians understand the results of potential interventions and design appropriate personalized care plans to reduce the risk of CKD.

## Discussion

**Fig. 5:**
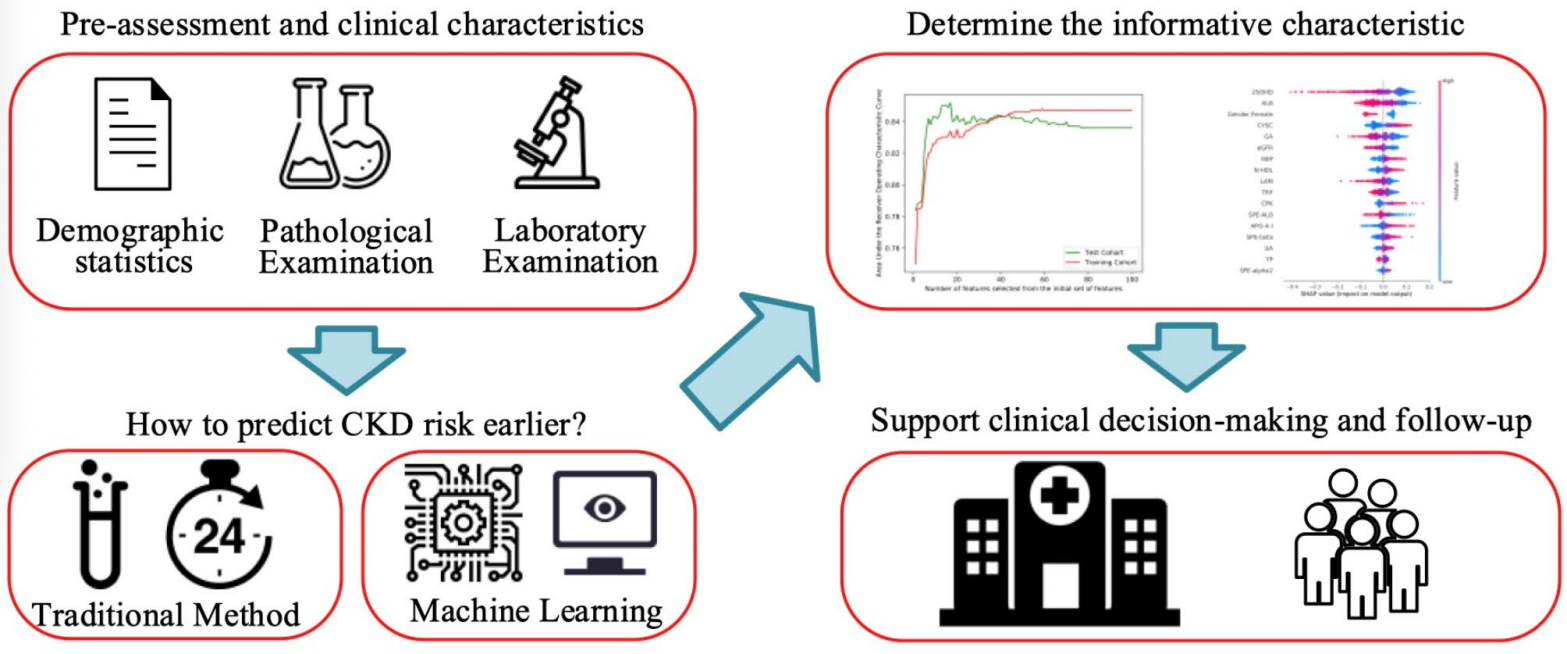
Overall summary of the study. Using common clinical variables, machine learning based approaches can effectively predict and explain the progression of CKD. Furthermore, decision support is provided for early intervention, and medical resource allocation is given for outpatients and those requiring a follow-up

The 24-h urine protein test has stringent patient compliance requirements and difficult follow-up procedures. The use of routine laboratory biochemical tests to replace the 24-h urine protein quantification will improve the convenience of outpatients and follow-up patients. In this retrospective cohort study, we developed machine learning algorithms using 100 easily obtainable clinical features for predicting CKD based on the severity of the proteinuria (Fig. 5). Some studies have shown that changes in the proteinuria are significantly associated with certain kidney function metrics, including a doubling of serum creatinine levels, rapid eGFR decline, and progression to end-stage kidney disease [25–27]. However, the detection of 24-h proteinuria is difficult owing to several factors, such as better applicability to hospitalized patients than to outpatients, poor patient compliance, and increased medical pressure. In the present study, the linear LR model exhibited the best AUC performance for single-model prediction, whereas the ensemble model (LR + XGBoost) exhibited the best AUC (0.856) among all models considered, with balanced specificity and sensitivity. Model fusion technology is therefore suitable for clinical decision support. Owing to the diversity of the available data and an adequate AUC performance, it can be concluded that the results of this study are informative for the rapid diagnostic identification of patients with CKD, with the mining of key risk factors contributing to subsequent treatment.

Artificial intelligence is being increasingly used in the medical field to predict various outcomes. Several longitudinal studies involving CKD have reported progress regarding the use of machine learning algorithms in CKD prediction. A survey by Huang et al. showed that 125 metabolites and 14 clinical variables can be used as predictors to establish a CKD prediction model for patients with type 2 diabetes (AUC = 0.857) [28]. Rashed-Al-Mahfuz et al. developed five models for predicting CKD using low-cost diagnostic screening. The RF accurately predicted (at a rate of 99.5%) patients at risk of CKD, but this high predictive power may be due to overfitting caused by too little data quantities [29]. Ferguson et al. also used routinely collected laboratory data and machine learning models to identify those at high risk of developing advanced CKD within the next 5 years [30]. However, none of these studies can provide personalized information for individual patients, thus hindering the ability of predictive models to support decision-making under clinical settings. This study provides a comprehensive framework for combining the predictive accuracy of CKD risk with interpretable results for the important characteristics of individual patients. Interestingly, consistent with the research by Xiao et al. on the use of proteinuria as a standard for CKD [31], the linear model achieved the best prediction performance in the prediction of multiple models; the ML model fusion used in this study can further improve the model AUC, which suggests that the model fusion scheme has potential practical capabilities. Similarly, some common factors such as ALB, TP, and eGFR have been found to be significantly related to CKD progression. More details about the above-mentioned studies are shown in Table S7. Particularly, our outcome differs from most existing reports, namely, we used 24-hour urinary protein as the outcome, while others were more based on eGFR, but the systemic changes in tubular creatinine secretion and extrarenal creatinine clearance could bias the results. Routinely, 24-hour urinary protein quantification is the gold standard for assessing the severity of CKD, but there are few studies that have stratified the risk of CKD with an outcome of 24-hour urinary protein, resulting in limited comparable studies, and this may be due to the difficulty in obtaining the results of 24-hour urinary protein quantification in clinical practice. For each patient, risk stratification of CKD and timely identification of high-risk are of great significance for rational allocation of limited clinical resources and treatment intervention. Our research has made up for these deficiencies to a certain extent.

Unlike many studies on CKD risk factors, we used RFE-LR algorithms to screen the most important variables that can be used for prediction and applied the SHAP values to explain the machine learning model. Based on the SHAP values, the 25OHD attribute was assigned the highest importance. The kidney is one of the main organs regulating vitamin D metabolism. The kidney internalizes 25OHD and converts 25(OH)D into 1,25(OH)2D. In CKD, the combination of a limited vitamin D intake and a reduced renal capacity to activate 25(OH)D into 1,25(OH)2D leads to a progressive vitamin D deficiency [32].Additionally, this study found that patients with 25OHD in the 28–35 nmol/L range require close monitoring to delay the progression of CKD. A close and regular review of 25OHD in such patients is recommended in clinical practice. However, further analysis of the actual health status of the patient is required to determine whether the dosing schedule of vitamin D can be adjusted. ALB was determined to be of the next highest importance based on the SHAP values; lower ALB levels are associated with the loss of kidney function. ALB accounts for approximately 60% of the total serum protein content, maintains colloidal osmotic pressure, and binds a variety of compounds under physiological conditions [33]. The glomerular filtration barrier prevents ALB from entering the ultrafiltrate. However, under the pathological condition of CKD, an increase in the effective radius of the barrier leads to protein loss, which further leads to a decrease in serum albumin levels [34]. The ALB of the point with zero SHAP values was approximately 36–37 g/L. This means that for patients with reduced renal function, even if the reference range for ALB is 35–55 g/L, ALB levels below 37 g/L may indicate moderate-to-severe renal impairment and require closer monitoring. Although the production rate of CYSC is more stable and its internal variability is smaller than that of Scr, there have been fewer studies on the renal function marker CYSC, which is a low-molecular-weight protein produced by nucleated cells at a constant rate and acts as lysosomal and cysteine proteases [35]. A recent meta-analysis showed similar findings; in particular, CYSC has a stronger correlation with renal function than Scr. We speculate that as an underlying explanation, CYSC is unaffected by muscle mass compared to Scr [36]. The interpretation based on the SHAP value is model-independent; that is, the SHAP value can be applied to different models. Therefore, although this research focused on CKD, the framework can be easily extended to the risk prediction and interpretation of other diseases to better support clinical decision-making.

Overall, in this study, an integrated framework for CKD risk prediction and interpretation is proposed to provide clinicians with decision support and model interpretation. Specifically, an integrated algorithm was developed to achieve a good prediction performance on the CKD dataset. While accurately predicting high-risk patients, it also achieves a strong interpretability for specific indicators. Finally, this study has certain limitations. Firstly, this is a single-center retrospective study, and there may be variations in the clinical characteristics of the data across different regions. Therefore, to assess the generalizability of the model, the conclusions drawn from this study need to be validated in external cohorts. Secondly, this study only considered the correlation between predictive factors and CKD, without considering causality. Thirdly, this study used conventional feature selection models, and the application of more recent advanced techniques such as NRF, AHEGFS, and BEFS may help identify more reliable CKD progression risk factors. Finally, the dataset used in this study only included blood-related indicators and ignored medical prescriptions and imaging examinations.

## Conclusions

In conclusion, we developed a machine learning model for predicting CKD based on proteinuria severity. The experimental results show that constructing a predictive interpretation framework can lead to a good predictive interpretation and provide effective clinical decision support. Another essential value is in providing new clinical insights for the management of patients requiring follow-up examinations for different diseases in large hospitals.

### Electronic supplementary material

Below is the link to the electronic supplementary material.

Supplementary Material 1

## Data Availability

The datasets used and/or analyzed during the current study are available from the corresponding author upon reasonable request.

## List of abbreviations

ROC: Receiver operating characteristic
AUC: Area under the curve
CKD: Chronic kidney disease
LR: Logistic regression
RF: Random forest
RFE-LR: Recursive feature elimination with logistic regression
SHAP: Shapley additive explanations
