# Supplementary material for: Risk factor mining and prediction of urine protein progression in chronic kidney disease: a machine learning- based study
Source: BMC Med Inform Decis Mak. 2023 Aug 31;23:173. doi: 10.1186/s12911-023-02269-2 (PMC10472702; doi:10.1186/s12911-023-02269-2)
Supplement: Supplementary file 1 — Supplementary Material 1 [file 12911_2023_2269_MOESM1_ESM.docx]

**Supplementary Material**

**Performance Evaluation Metrics**

In the current study, the following metrics were applied to evaluate the performance of each model.

i.) TP (true positive), The prediction is correct and positive.

ii.) FP (false positive), The prediction is wrong and positive.

iii.) TN (true negative), The prediction is correct and negative

iv.) FN (false negative), The prediction is wrong and negative.

v.) Confusion Matrix, an error matrix used to visually evaluate the performance of supervised learning algorithms. (Table S1)

vi.) Accuracy, which refers to the proportion of correctly predicted samples to the total number of samples (Eq.1)

| $Accuracy=\frac{TP+TN}{TP+FP+TN+FN}$ | (1) |
| --- | --- |

vii.) TPR (true positive rate), or Sensitivity (Eq.2)

| $TPR=Recall=Sensitivity=\frac{TP}{TP+FN}$ | (2) |
| --- | --- |

viii.) FPR (false positive rate), or type I error probability (Eq.3)

| $FPR=FP/(FP+TN)$ | (3) |
| --- | --- |

ix.) TNR (true negative rate), or Specificity (Eq.4)

| $Specificity=TNR=\frac{TN}{TN+FP}=1-FPR$ | (4) |
| --- | --- |

x.) Precision (Eq.5)

| $Precision=\frac{TP}{TP+FP}$ | (5) |
| --- | --- |

xi.) ROC (Receiver Operating Characteristic) curve, a curve determined by TPR and FPR for evaluating the model performance.

xii.) AUC (Area Under Curve), an index used to evaluate the model's performance in distinguishing positive samples from negative samples.

xiii.) AP (Average precision), averaging the accuracy at different recall points. (Eq.6)

| $AP=\sum_{n} (R_{n}-R_{n-1})P_{n}$ | (6) |
| --- | --- |

The coordinates of the nth threshold are *P_n_* (precision) and *R_n_* (recall).

**Model establishment and brief illustrations**

i.) Least absolute shrinkage and seletion operator, Lasso: Lasso can get a more refined model by constructing a penalty function, so that it can compress some regression coefficients to achieve the purpose of screening features. (Eq.7)

| $\min_{\beta_{0},\beta}\left\{ \sum_{i=1}^{N} \left( y_{i}-\beta_{0}-x_{i}^{T}\beta\right)^{2} \right\}subject to \sum_{j=1}^{p} \left\vert\beta_{j} \right\vert\leq t$ | (7) |
| --- | --- |

Where $\beta_{0}$ is the constant coefficient, $\beta$ is the coefficient matrix of the variable, and $t$ is the penalty term, which is used to control the complexity of the model. Define X as the variable expression matrix.

ii.) XGBoost: This gradient boosting classifier is based on boosted trees, which objective function includes loss function and regular term.

iii.) GaussianNB：GNB assumes that the observations whose features belong to a certain class conform to a Gaussian distribution.

iv.) Neural network, NN: Based on the error between the calculated output and the target output, improving the weights is the core of the neural network's work.

v.) Ridge regression, RR: RR is a multiple linear regression which integrates the two norm regularization into the least squares model [4]. (Eq.8)

| $(\alpha,\beta)=\mathrm{argmin}(\sum_{i=1}^{n} (y_{i}-\alpha_{i}-X_{i}\beta)^{2}+\lambda\vert\vert\beta\vert\vert_{2}^{2})$ | (8) |
| --- | --- |

Where $X_{i}$ is the $i$_th_ independent variable, $\alpha$and $\beta$ are regression coefficient, $||\beta||_{2}$ stand for two norm , $y_{i}$ is the outcome value of $X_{i}$, $n$ is the number of sample used in the model, $\lambda$is the penalty parameter.

vi.) Logistic regression, LR: This is a general regression model：(Eq.9)

| $Logit(P)=\alpha+\beta_{i}X_{i}$ | (9) |
| --- | --- |

Where $\alpha$is a constant, $\beta_{i}$ stand for regression coefficient, $X_{i}$ is the $i$_th_ independent variable, P stands for random sampling of possible outcomes.

**Model pseudo-code and brief descriptions**

i). Synthetic minority oversampling, SMOTE: The SMOTE algorithm can be used to oversample the minority class in a dataset by generating synthetic samples that are similar to the original minority class samples. The algorithm is simple and effective and has been widely used in various classification tasks. The implementation of this study is given in the pseudo-code of Algorithm 1.

| Algorithm 1. |
| --- |
| **Inputs**: |
| X - input feature matrix, shape=(n_samples, n_features) |
| y - input label, shape=(n_samples,) |
| k_neighbors - number of nearest neighbors to be selected |
| n_samples - number of new samples to be generated |
| **Outputs**: |
| X_new - newly generated feature matrix, shape=(n_samples, n_features) |
| y_new - newly generated labels, shape=(n_samples,) |
| Steps: |
| 1. Compute the k_neighbors nearest neighbors for each sample and record their indices.   1. For each sample *X[i]*, compute its distance to other samples and find k_neighbors samples that are closest to it. 2. Store the indices of these k_neighbors samples in a list *K[i]*. |
| 2. For each sample *X[i]*, randomly select one of its k_neighbors, say *X[j]*.   1. Compute the difference between *X[j]* and *X[i]* and multiply it by a random number between 0 and 1. 2. Add the resulting vector to *X[i]* to obtain a new synthetic sample, *X_new[k]*. 3. Assign the label of *X[i]* to *X_new[k]*, i.e., *y_new[k]* = *y[i]*. |
| 3. Repeat step 2 for n_samples times to generate n_samples synthetic samples. |
| 4. Combine the original samples and the newly generated samples to obtain the final feature matrix X_new and the final label vector y_new. |

ii.) Recursive feature elimination with logistic regression, RFE-LR: RFE-LR is a common method in packaging feature selection. Based on the iterative method, the RFE method constantly removes the features with the lowest contribution score, and then evaluates the model score in each cycle to obtain the optimal evaluation feature subset. The implementation of this study is given in the pseudo-code of Algorithm 2.

| Algorithm 2. |
| --- |
| **Inputs**: |
| Training set *T*_s_ |
| Set of p features *F*_e_ = {*f*_1_, … , *f_n_*} |
| Ranking method *M_e_ (T_s_,, F_e_)* |
| **Outputs**: |
| Final ranking *R* |
| Steps: |
| 1. Repeat for *i* in {1: *n*} |
| 2. Rank set *F_e_* using M*_e_* (*T*_s_, *F*_e)_ |
| 3. Best ranked feature in *F_e_* |
| 4. Extract the best index set from *R* |

iii.) Ensemble model: The ensemble method is mainly based on the overall construction by combining multiple estimation models to improve the generalization/robustness of a single classifier. The weighted Average (soft voting) is used for model fusion in this study and the pseudo-code is Algorithm 3.

| Algorithm 3. |
| --- |
| **Inputs**: |
| The optimal classifier *C*_s_ |
| Set of voting weight *V*_e_ = {*V*_1_, … , *V_n_*} |
| Ranking method *M_e_ (C_s_,, V_e_)* |
| **Outputs**: |
| Final ensemble model |
| Steps: |
| 1. Repeat for *i* in {1: *n*} |
| 2. Voting weight set *V_e_* using M*_e_* (*C*_s_, *V*_e)_ |
| 3. Best ranked method in *V_e_* |
| 4. Obtained the best ensemble model |

**Supplementary figure**

**
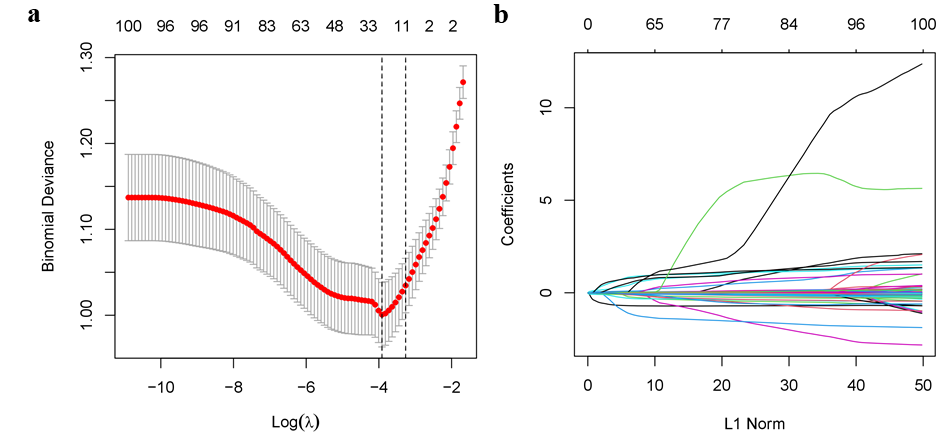
**

Figure S1. LASSO feature subset construction. (a) Coefficient profiles and identification of the best parameter (lambda) in the least absolute shrinkage and selection operator. (b) The 21 features retained to construct the optimal model.

**Supplementary tables**

**Table S1. Confusion matrix**

| Confusion matrix | | Prediction | |
| --- | --- | --- | --- |
|  |  | Negative | Positive |
| Actural | Negative | TN | FP |
|  | Positive | FN | TP |

**Table S2. Feature information**

| **Item** | **Explain** |  |  |
| --- | --- | --- | --- |
| **Demographic** |  |  | |
| Gender | Gender |  | |
| Age | Age |  | |
| **Liver function** |  |  | |
| DBIL | Direct bilirubin |  | |
| TP | Total protein |
| ALB | Albumin |
| GLO | Globulin |
| AGRatio | Albumin/Globulin |
| SPE.ALB | Serum protein electrophoresis. albumin |
| SPE.alpha1 | Serum protein electrophoresis. alpha1 |
| SPE.alpha2 | Serum protein electrophoresis. alpha2 |
| SPE.beta | Serum protein electrophoresis. beta |
| SPE.gamma | Serum protein electrophoresis. gamma |
| AST | Aspartate transaminase |
| ALP | Alkaline phosphatase |
| GGT | Glutamyltransferase |
| TBA | Determination of serum bile acids |
| APLB1 | Prealbumin |
| **Cardiac makers** |  |  | |
| ALT | alanine aminotransferase |  | |
| LDH | Lactic dehydrogenase |  | |
| **Renal function** |  |  | |
| BUN | blood urea nitrogen |
| CRE | Serum creatinine |
| eGFR | Estimate glomerular filtration rate |
| CYSC | Cystatin C |
| **Purine metabolism** |  |  | |
| UA | Uric acid |  | |
| **Glycometabolism** |  |  | |
| GA | Glycated albumin |  | |
| **Lipid metabolism** |  |  | |
| TG | Triglycerides |
| LDL | Low density lipoprotein |
| N.HDL | Non high density lipoprotein |
| APO.A.I | Apolipoprotein A |
| APO.B | Apolipoprotein B |
| APO.E | Apolipoprotein E |
| B2M | β2-Microglobulin |  | |
| LPA1 | Lipase |
| **Blood electrolytes** |  |  | |
| Na | Sodium |
| K | Potassium |
| Cl | Chloridion |
| CO2 | Carbon dioxide |
| AG | Anion gap |
| Ca | Calcium |
| P | Phosphorus |
| Mg | Magnesium |
| **Metabolism** |  |  | |
| CPK | Creatine phosphokinase |
| CK.MB | MB isoform of creatine kinase |
| CK.MM | MM isoform of creatine kinase |
| RBP | Retinol conjugated protein |  | |
| HCY | Homocysteine |
| **Inflammatory** |  |  | |
| CRP | C-reactive protein |  | |
| **Markers of anemia** |  |  | |
| IRON | Serum iron |
| UIBC | Unsaturated iron binding force |
| TIBC | Total iron binding capacity |
| TRF | Transferrin |  | |
| RF | Rheumatoid factor |  | |
| TDB | Ferroprotein |
| B12 | Vitamin B12 |
| FOL | Folic acid |
| TS. | Transferrin saturation |
| **Autoantibody** |  |  | |
| IGG | Immunoglobulin G |
| IGA | Immunoglobulin A |
| IGM | Immunoglobulin M |
| ASO | Antistreptolysin |  | |
| KAP | Kappa light chain |  | |
| LAM | Lambda light chain |  | |
| IGE | Immunoglobulin E |
| **Immune** |  |  | |
| C3 | Complement 3 |
| C4 | Complement 4 |
| CH50 | Serum total complement |
| **Tumor marker** |  |  | |
| CA199 | Carbohydrate antigen 199 |
| NSE | Neuron-specific enolase |
| **Thyroid function** |  |  | |
| T3 | Total triiodothyronine 3 |
| T4 | Total triiodothyronine 4 |
| FT3 | Free triiodothyronine 3 |
| FT4 | Free triiodothyronine 4 |
| TSH | Thyrotropic hormone |
| PTH | Parathyroid hormone |
| **Bone metabolic** |  |  | |
| NTX | Osteocalcin |
| 25OHD | 25 Hydroxyvitamin D |
| **Blood routine** |  |
| HGB | Hemoglobin |
| HCT | Haematocrit |
| MCV | Mean corpuscular volume |
| MCH | Mean corpuscular hemoglobin |
| MCHC | Mean corpuscular-hemoglobin concen |
| PLT | Platelet count |
| WBC | White blood cell |
| NEUT. | Neutrophilic granulocyte |
| LYMPH. | Lymphocyte |
| MONO. | Monocyte |
| EO. | Eosinophils |
| BASO. | Basophilic granulocyte |
| NEUT% | Neutrophilic granulocyte percentage |
| LYMPH% | Lymphocyte percentage |
| MONO% | Monocyte percentage |
| EO% | Eosinophils percentage |
| BASO% | Basophilic granulocyte percentage |
| RDW.CV% | Coefficient of variation of RBC volume size percentage |
| RDW.SD% | Standard deviation of erythrocyte distribution width percentage |
| MPV% | Mean platelet volume percentage |
| PCT% | procalcitonin percentage |
| P.LCR | Proportion of platelets |
| PDW | Distribution width of platelets |
| RET. | Reticulocyte |
| **Renal function** |  |
| U.PRO | 24-hour urinary protein quantity |

**Table S3 Performance summary of all features.**

| Models | AUC | 95%CI | | sensitivity | specificity | accuracy | AP |
| --- | --- | --- | --- | --- | --- | --- | --- |
|  |  | Lower bound | Upper bound |  |  |  |  |
| XGBoost | 0.828 | 0.78 | 0.876 | 0.702 | 0.813 | 0.739 | 0.914 |
| GNB | 0.769 | 0.711 | 0.827 | 0.746 | 0.725 | 0.739 | 0.863 |
| NN | 0.772 | 0.712 | 0.832 | 0.746 | 0.714 | 0.735 | 0.849 |
| Ridge | 0.816 | 0.764 | 0.868 | 0.691 | 0.835 | 0.739 | 0.899 |
| LR | **0.833** | 0.785 | 0.882 | 0.785 | 0.747 | 0.772 | 0.916 |

**Table S4 Performance summary after Lasso feature screening.**

| Models | AUC | 95%CI | | sensitivity | specificity | accuracy | AP |
| --- | --- | --- | --- | --- | --- | --- | --- |
|  |  | Lower bound | Upper bound |  |  |  |  |
| XGBoost | 0.812 | 0.76 | 0.864 | 0.669 | 0.824 | 0.721 | 0.895 |
| GNB | 0.776 | 0.72 | 0.832 | 0.735 | 0.758 | 0.743 | 0.883 |
| NN | 0.793 | 0.736 | 0.85 | 0.762 | 0.769 | 0.765 | 0.865 |
| Ridge | 0.822 | 0.773 | 0.871 | 0.812 | 0.714 | 0.779 | 0.912 |
| LR | **0.828** | 0.778 | 0.877 | 0.79 | 0.747 | 0.776 | 0.911 |

**Table S5 Performance summary before the hyper-optimization.**

| Models | AUC | 95%CI | | sensitivity | specificity | accuracy | AP |
| --- | --- | --- | --- | --- | --- | --- | --- |
|  |  | Lower bound | Upper bound |  |  |  |  |
| XGBoost | 0.824 | 0.774 | 0.873 | 0.685 | 0.835 | 0.735 | 0.906 |
| GNB | 0.808 | 0.755 | 0.861 | 0.751 | 0.791 | 0.765 | 0.893 |
| NN | 0.783 | 0.728 | 0.838 | 0.779 | 0.681 | 0.746 | 0.883 |
| Ridge | 0.835 | 0.787 | 0.883 | 0.779 | 0.78 | 0.779 | 0.917 |
| LR | **0.839** | 0.792 | 0.886 | 0.751 | 0.835 | 0.779 | 0.917 |

**Table S6 Results of running time of different machine learning algorithms**

| Model | Training cohort of each hyper-parameter(s) | Test cohort(s) |
| --- | --- | --- |
| XGBoost | 4.647±0.091 | 0.001±0.000 |
| GNB | 0.011±0.001 | 0.002±0.002 |
| NN | 3.422±0.307 | 0.001±0.000 |
| Ridge | 0.272±0.049 | 0.001±0.000 |
| LR | 0.222±0.016 | 0.003±0.002 |
| Ensemble | 1.833±0.017 | 0.002±0.001 |

**Table S7 Results of comparison with other literature on the prediction of CKD**

| PMID | Author | Year | Outcome definition | Data quantities | Feature screening method | Feature interpretation | AUC |
| --- | --- | --- | --- | --- | --- | --- | --- |
| This study | Lu et al. | / | 24-hour urine protein | 1,358 | RFE-LR and Lasso algorithm | SHAP algorithm | 0.856 |
| 30971285 | Xiao et al. | 2019 | 24-hour urine protein | 551 | N/A | N/A | 0.873 |
| 33024004 | Huang et al. | 2020 | eGFR and UACR | 1,838 | priority-Lasso and multivariate logistic regression | N/A | 0.857 |
| 33948393 | Rashed-Al-Mahfuz et al. | 2021 | eGFR | 400 | N/A | SHAP algorithm | 0.990 |
| 35967110 | Ferguson et al. | 2022 | eGFR | 77,196 | N/A | N/A | 0.880 |
